# The Frequency of Apoplexy among the Higher Classes, with Suggestions for Its Prevention and Escape from Fatality

**Published:** 1898-05

**Authors:** Elmer Lee


### THE FREQUENCY OF APOPLEXY AMONG THE HIGHER CLASSES, WITH SUGGESTIONS FOR ITS PREVENTION AND ESCAPE FROM FATALITY.

BY ELMER LEE, A. M., M. D., Ph.d.

It is rare that an animal dies of apoplexy, but man's days are frequently ended by this disease, coming upon him generally later than the age of 50. The contrast between the simple life of the animal and the complex one of the man is in favor of the former, so far as it relates to health. At the beginning, the young animal and the child are on the same natural footing; both are nature's sweet and innocent children. Little by little the child grows away from dependence upon natural methods for its guide, substituting the artificial as life advances and ever becomes more complex. But the animal pursues an even tenor of regularity and simplicity, avoids the excesses and escapes the sad consequences of sickness, pain and premature death.

The beginning of disease is at that moment when the laws of health are first violated, and starts with infancy and childhood in the majority of cases. The great love of the parent for the offspring gradually yields to the desires of the young, even though inconsistent with reason and the gratification of them detrimental to its best present and future welfare. In the young vitality is vigorous, and in spite of bad care and over-indulgence from the well-meaning parents, it carries a large proportion of the children to adult age and beyond. The early mistake in the training of the child is the principal factor that develops into the disaster of the later years. When the child comes into the world it is ignorant of the requirements necessary for life and health, and is at the mercy of conditions beyond its power to change with reference to those who shall conduct the young life safely or to its destruction. The laws relating to preservation of health being so little comprehended and so much neglected, affords just the state of things suited to start the beginner in life into the wrong path. The early impressions, very hard to correct later in life, added to misapprehension and error in the process of growth, lay the certain foundations for disease and premature fatality.

Each nationality has its peculiar domestic customs which are transmitted to the offspring, and while some of these habits of life are innocent, many of them are directly fatal in due course. The German teaches the infant to cultivate a taste for mild liquor of the nature of wine and beer. The youth is encouraged to drink the health of the fatherland, while the adult saturates every cell and tissue in the body with a four per-cent. solution of alcohol in malt, with supposed security and a conscious feeling of superiority to his neighbor who tipples with high wine and whisky. The drinker of the weaker beverage makes up in quantity what his favorite drink lacks in strength as compared with the stronger poison of French or English friends. The result in all cases is the same, provided enough is taken and for sufficient time to destroy vital processes.

The American is the jolly associate and imitator of the convivial foreigner, freely indulging in his virtues and vices, and equal to him in the consumption of potations both mild and strong. Familiarity with the titles and literature pertaining to alcoholic poisons, otherwise known in polite circles as "cocktails," "ginslings," "brandy-smashes," "Tom and Jerry," and "nightcaps," together with a very long list of fantastic names supposed to represent some peculiar virtue, forsooth, which is good for man, is early acquired by the youth who are to constitute later the apoplectic surprises which are noted in the daily morning paper, thereby forming a morsel for comment even by those who are in the same danger but heed it not. In natural health the ideal desire is to prolong life and to walk in pleasant paths. Every one hopes to escape pain and sickness, but of the many who so desire a few only succeed. Nature is most gentle and tender, but at the same time absolutely just, and what is suffered by man is through either folly or ignorance in his blind eagerness to attain the satisfaction of a bauble.

So long as the grape grows and the fermented juice is brought habitually in contact with the delicate cells of a human body, and while human food is mixed with acidulated alcohol in the form of frothing beer, that man who loves the solace of these potations, and who persists in the mistake that he is stimulated and thereby refreshed, is sure to fall a fatal itvimc to an implacable fate which patiently waits and finally seals hs doom. There are many who belong to this unfortunate class that escape apoplexy to fall earlier in life from maladies with different names, but hastened and effectual in their fatality, from even that abuse of the alcoholic influence disguised under the fair apology of moderation. With an error of life of such wide and extended daily use as that of habitual drinking of liquor, is there anything strange in frequent fatalities due to apoplexy?

The number of fatalities from apoplexy would be even far greater if it were not that death claims many victims of wrong living ere the course of mistakes run by weak and deluded humanity reaches the apoplectic goal. This is a frequent disease among the higher classes, for it is in this class that those excesses which contribute to produce apoplexy prevail to such a degree as to keep the press supplied with obituaries of those accounted distinguished and possessed of lovable characters.

Apoplexy refers to an accident to an artery in the brain resulting in intracranial hemorrhage and pressure, causing sudden loss or diminution of sensation and power of voluntary motion. The artery ruptures through inherent weakness of the wall from previous disease. The accident is likely to be fatal, but there may be one or several strokes or ruptures at varying intervals before the patient perishes. The rupture of an intracranial artery, if near a vital nerve center, rapidly brings the life which has lived in disobedience to nature to an abrupt termination, but if the artery ruptured is somewhat remote, protracted paralysis, more or less complete, is the result.

In the light of the experience of yesterday, it would be possible to pass the earthly days without apoplexy save in most rare instances, were the wisdom heeded which is gained by the knowledge that not one who has lived in violation to natural law has escaped a penalty exactly in proportion to his transgression. So intense is the demand for artificial stimulation when once the natural appetite is perverted by thoughtless and innocent indulgences, later by fierce and uncontrolled intemperance in eating, drinking and sinning, in desecration of the holy temple of health, that life without these health-destroying agents seems to such characters a failure. The use of artificial and false doctrines poisons in gradual degree the whole race. The American teaches the child to prepare for fatty degeneration and its consequent choice collections of bodily afflictions, by setting examples that if followed lead to destruction of health and loss of life.

The coffee-pot is high ruler of the table and the digestion of the American family, and dyspepsia prevails to the consternation of all classes and of nearly every age. The sauces and vinegars, the relishes and dressings, the salads and sweets, innocent in themselves but habitually used and abused, poison the nourishment of the body, enfeeble the circulation and weaken the vital resistance. The source of weakness as well as that of strength is regulated by the stomach, and in this organ arise the initial causes that tend to sickness and death of the body. As has been truly stated by Dr. Page of Boston, "dyspepsia is incipient consumption," thus it may be also clearly proven that from the same source arises that condition which finally ends life in the apoplectic stroke.

Too much to eat is a misfortune which has for its penalty lack of digestion and failure to relish with a keen satisfaction the whole blessing that food is intended to confer; while to the multitude, who have sufficient food, the hardship would not be a misfortune so great if the knowledge of food and its best use was better understood and practiced. One class has too much and the other too little, and to both come disease, for in each case the natural laws of health are violated in the selection of the articles of diet and in the preparation which civilized cookery has visited upon the members of the human family.

The almost universal custom of three meals a day finds favor among physicians and the laity alike, and even in the treatment of sickness when the system is embarrassed and struggling to overcome the obstacles forced into the unwilling human machine against the prolonged remonstrances of the digestive organs. Through excessive feeding and insufficient elimination the load ever increases, and when nature is outraged to the limit of forbearance, mild protest is succeeded by partial or complete failure to proceed further. Strange as it may be, and frequently by advice of the professional mind, the very cause of the sickness is kept up by an amount of food, and of an improper character, which, while it would not amount to a surfeit to the well, does further interfere with a rapid restoration to health, and is not infrequently a direct factor in stretching a slight matter into a long sickness with recurring relapses and death.

The hard out-of-door laborer, swinging his pick and working with the shovel, is entitled to three meals a day, for they are earned and likewise needed. But this class does not constitute the apoplectic victims. This advantage, if balanced with temperance among the wage-earners, would augment their happiness and add years to life and minimize their sorrows. Correct information regarding the essentials of eating and drinking for the sustenance and comfort of health has and ever will stand as the first requisite toward the prevention of sickness and apoplexy, and while it is either misunderstood or neglected, life will be robbed of its sweets to the individual, and society of lovable and useful characters. Two moderate meals of natural and healthful food is the limit that constitutes safety to the higher classes. A morning and evening meal, with bread and fruit for the midday refreshment, with water instead of artificial drinks, would spare the waste of good friends and distinguished public men, a class generally at the mercy of fashion in eating and to the quickening cup with injury in the drop and fatality in excess. That person who has any symptom related to the digestive organs needs advice of the highest order, and if the case progresses toward a worse condition it would be far better to seek counsel from a competent medical adviser, and never from the plausible and cunningly-worded falsehoods for whom newspaper and almanac cures are invented.

Careful experiments with different materials and weights used as clothing for the human body have established that the modern dress of heavy flannel beneath the woolens outside, with additional accessories to dress, even in cold weather, is in excess of the requirements. Then how much more unnatural is it to wear more than is needed in mild and warm climates, and such as that which includes New York and Philadelphia? By dressing the body with too much clothing, dangers to health are increased and recovery from chronic diseases retarded. As a plant would soon die without its trunk and branches freely exposed to air and light, so the human body dies gradually, though one of the causes is often overlooked or attributed to other explanations. The best light-weight underwear procurable in either silk, cotton or linen mesh for the youth and the adult, in health and sickness, is indicated both in winter and summer. Flannels are no longer recommended, for scientific reasons.

Then with a correct food supply and the use of pure water for the drink, freely taken all through life, together with right use of covering for the body, which will admit of enough ventilation (seldom found among my patients and the sick generally), with sound medical counsel, places it within the reach of the average individual to avoid a premature fatality. Open-air exercise is indispensable to the preservation of health and to the prevention of apoplexy. The causes of sickness and disease are the causes of apoplexy, and prevention and escape from fatality means that the individual can not violate natural laws of organization and vitality, with coarse jeer at health requirements. To the hourly and daily and non-compromising demands to practice wisdom, there is no appeal, and the penalty for violations of nature's code for man's use is paid with a forfeit of his life.—*The Journal.*

